# Supplementary material for: Role and mechanism of NCAPD3 in promoting malignant behaviors in gastric cancer
Source: Front Pharmacol. 2024 Apr 22;15:1341039. doi: 10.3389/fphar.2024.1341039 (PMC11070777; doi:10.3389/fphar.2024.1341039)
Supplement: Supplementary file 11 [file DataSheet2.ZIP › GSEA/immunologic/my_analysis.Gsea.1599462003817/GSE18791_CTRL_VS_NEWCASTLE_VIRUS_DC_6H_UP.html]

Details for gene set GSE18791\_CTRL\_VS\_NEWCASTLE\_VIRUS\_DC\_6H\_UP[GSEA]

|  || Dataset | filtered\_dataset.sample\_info.cls#WT\_versus\_NCAPD3\_MUT |
| Phenotype | sample\_info.cls#WT\_versus\_NCAPD3\_MUT |
| Upregulated in class | WT |
| GeneSet | GSE18791\_CTRL\_VS\_NEWCASTLE\_VIRUS\_DC\_6H\_UP |
| Enrichment Score (ES) | 0.37955594 |
| Normalized Enrichment Score (NES) | 1.9278054 |
| Nominal p-value | 0.0050167223 |
| FDR q-value | 0.7180064 |
| FWER p-Value | 1.0 |
Table: GSEA Results Summary

  

Fig 1: Enrichment plot: GSE18791\_CTRL\_VS\_NEWCASTLE\_VIRUS\_DC\_6H\_UP      
 Profile of the Running ES Score & Positions of GeneSet Members on the Rank Ordered List

  

| SYMBOL | TITLE | RANK IN GENE LIST | RANK METRIC SCORE | RUNNING ES | CORE ENRICHMENT || 1 | 64172 | OSGEPL1 | 6 | 1.152 | 0.0556 | Yes |
| 2 | 222236 | NAPEPLD | 36 | 0.977 | 0.0854 | Yes |
| 3 | 80746 | TSEN2 | 53 | 0.909 | 0.1211 | Yes |
| 4 | 55794 | DDX28 | 68 | 0.856 | 0.1555 | Yes |
| 5 | 153364 | MBLAC2 | 79 | 0.838 | 0.1919 | Yes |
| 6 | 55900 | ZNF302 | 145 | 0.740 | 0.1832 | Yes |
| 7 | 11162 | NUDT6 | 162 | 0.716 | 0.2089 | Yes |
| 8 | 57325 | CSRP2BP | 176 | 0.698 | 0.2358 | Yes |
| 9 | 5189 | PEX1 | 200 | 0.676 | 0.2543 | Yes |
| 10 | 116236 | ABHD15 | 203 | 0.672 | 0.2879 | Yes |
| 11 | 55172 | DNAAF2 | 224 | 0.657 | 0.3075 | Yes |
| 12 | 403313 | PLPP6 | 274 | 0.615 | 0.3040 | Yes |
| 13 | 84078 | KBTBD7 | 295 | 0.594 | 0.3204 | Yes |
| 14 | 92342 | METTL18 | 322 | 0.579 | 0.3317 | Yes |
| 15 | 9786 | KIAA0586 | 379 | 0.547 | 0.3195 | Yes |
| 16 | 79896 | THNSL1 | 407 | 0.534 | 0.3277 | Yes |
| 17 | 54920 | DUS2 | 509 | 0.470 | 0.2788 | Yes |
| 18 | 9816 | URB2 | 581 | 0.434 | 0.2498 | Yes |
| 19 | 93134 | ZNF561 | 590 | 0.431 | 0.2664 | Yes |
| 20 | 51388 | NIP7 | 593 | 0.428 | 0.2873 | Yes |
| 21 | 23404 | EXOSC2 | 609 | 0.418 | 0.2981 | Yes |
| 22 | 5147 | PDE6D | 624 | 0.410 | 0.3093 | Yes |
| 23 | 55735 | DNAJC11 | 625 | 0.409 | 0.3306 | Yes |
| 24 | 2021 | ENDOG | 648 | 0.400 | 0.3355 | Yes |
| 25 | 56252 | YLPM1 | 665 | 0.394 | 0.3444 | Yes |
| 26 | 23212 | RRS1 | 681 | 0.387 | 0.3537 | Yes |
| 27 | 3145 | HMBS | 682 | 0.386 | 0.3738 | Yes |
| 28 | 84975 | MFSD5 | 702 | 0.376 | 0.3796 | Yes |
| 29 | 6182 | MRPL12 | 729 | 0.359 | 0.3794 | No |
| 30 | 26608 | TBL2 | 821 | 0.304 | 0.3291 | No |
| 31 | 8738 | CRADD | 922 | -0.349 | 0.2747 | No |
| 32 | 11007 | CCDC85B | 953 | -0.373 | 0.2723 | No |
| 33 | 1628 | DBP | 1029 | -0.429 | 0.2402 | No |
| 34 | 861 | RUNX1 | 1293 | -0.687 | 0.0850 | No |
Table: GSEA details [plain text format]

  

Fig 2: GSE18791\_CTRL\_VS\_NEWCASTLE\_VIRUS\_DC\_6H\_UP      
 Blue-Pink O' Gram in the Space of the Analyzed GeneSet

  

Fig 3: GSE18791\_CTRL\_VS\_NEWCASTLE\_VIRUS\_DC\_6H\_UP: Random ES distribution      
 Gene set null distribution of ES for **GSE18791\_CTRL\_VS\_NEWCASTLE\_VIRUS\_DC\_6H\_UP**
